# Development and Biocompatibility of Collagen-Based Composites Enriched with Nanoparticles of Strontium Containing Mesoporous Glass

**DOI:** 10.3390/ma12223719

**Published:** 2019-11-11

**Authors:** Giorgia Montalbano, Giorgia Borciani, Carlotta Pontremoli, Gabriela Ciapetti, Monica Mattioli-Belmonte, Sonia Fiorilli, Chiara Vitale-Brovarone

**Affiliations:** 1 Applied Science and Technology Department, Politecnico di Torino, Corso Duca degli Abruzzi 24, 10129 Torino, Italy; giorgia.montalbano@polito.it (G.M.); giorgia.borciani@polito.it (G.B.); carlotta.pontremoli@polito.it (C.P.); sonia.fiorilli@polito.it (S.F.); 2 Laboratorio di Fisiopatologia Ortopedica e Medicina Rigenerativa, IRCCS Istituto Ortopedico Rizzoli, 40136 Bologna, Italy; gabriela.ciapetti@ior.it; 3 Dipartimento di Scienze Cliniche e Molecolari, DISCLIMO, Università Politecnica delle Marche, 60100 Ancona, Italy; m.mattioli@univpm.it

**Keywords:** mesoporous bioactive glasses, type I collagen, 4-StarPEG, collagen chemical crosslinking, strontium release, biomimetic composites, bone tissue engineering

## Abstract

In the last years bone tissue engineering has been increasingly indicated as a valid solution to meet the challenging requirements for a healthy bone regeneration in case of bone loss or fracture. In such a context, bioactive glasses have already proved their great potential in promoting the regeneration of new bone tissue due to their high bioactivity. In addition, their composition and structure enable us to incorporate and subsequently release therapeutic ions such as strontium, enhancing the osteogenic properties of the material. The incorporation of these inorganic systems in polymeric matrices enables the formulation of composite systems suitable for the design of bone scaffolds or delivery platforms. Among the natural polymers, type I collagen represents the main organic phase of bone and thus is a good candidate to develop biomimetic bioactive systems for bone tissue regeneration. However, alongside the specific composition and structure, the key factor in the design of new biosystems is creating a suitable interaction with cells and the host tissue. In this scenario, the presented study aimed at combining nano-sized mesoporous bioactive glasses produced by means of a sol–gel route with type I collagen in order to develop a bioactive hybrid formulation suitable for bone tissue engineering applications. The designed system has been fully characterized in terms of physico-chemical and morphological analyses and the ability to release Sr^2+^ ions has been studied observing a more sustained profile in presence of the collagenous matrix. With the aim to improve the mechanical and thermal stability of the resulting hybrid system, a chemical crosslinking approach using 4-star poly (ethylene glycol) ether tetrasuccinimidyl glutarate (4-StarPEG) has been explored. The biocompatibility of both non-crosslinked and 4-StarPEG crosslinked systems was evaluated by in vitro tests with human osteoblast-like MG-63 cells. Collected results confirmed the high biocompatibility of composites, showing a good viability and adhesion of cells when cultured onto the biomaterial samples.

## 1. Introduction 

The increasing age of the world population is associated to the growing demand for the treatment of bone defects derived from aging and pathological conditions such as osteoporosis [1,2,3]. The need for healthy bone regeneration therapy has increased the interest in bone tissue engineering as valid alternative to the currently used pharmaceutical and surgical approaches for bone repair, which are frequently related to several limitations and failures [2,4,5,6].

Bone tissue engineering aims at designing biomaterials and scaffolds able to guide and promote the regeneration of healthy and functional tissue, taking inspiration from the native structure and composition of bone [7,8,9,10].

The use of bioactive glasses in bone tissue engineering has been frequently reported and proved successful outcomes in bone regeneration treatments [11,12]. Their peculiar ability to promote hydroxyapatite deposition in presence of physiological fluids allows them to form strong interfacial bonds with bone tissue [11,13]. Traditional sol–gel synthesis methods combined with the use of templating agents enable the creation of mesoporous bioactive glasses (MBG) with even increased bioactive properties due to the higher surface area due to their nanoporosity [13]. Several in vivo studies also reported that the presence of strontium is associated to a significant enhancement of new bone formation and a reduction of osteoclast resorptive activity [13,14,15]. Sr^2+^ containing bioactive glasses have been thus widely proposed as therapeutic agents in case of bone disease such as osteoporosis due to their ability to release calcium and strontium cations [16,17]. 

Given the nanocomposite nature of bone and with the aim to develop materials with enhanced biomimicry, MBGs can be combined with type I collagen, which represents the main organic phase of native bone tissue [4,11,12,18,19].

The main challenge in the design of suitable biomaterials for bone tissue engineering mainly relates to the realization of stable and biocompatible structures able to promote a positive crosstalk between material and cells. Most of the developed cell-responsive hybrid systems for bone tissue engineering applications combine bioactive inorganic phases with functionalized polymers or a blend of naturally derived and synthetic polymers, without actually reproducing the native environment both in terms of compositional and nanostructural properties [10,12,20]. 

On the contrary, type I collagen matrices are considered an ideal template for bone tissue engineering since they reproduce a biological environment similar to the native extracellular matrix [4,21,22]. Collagen molecules are able to self-assemble creating a nanofibrous matrix upon physiological conditions and provide a valid support for cell adhesion, proliferation and differentiation. However, reconstituted forms of collagen normally lack sufficient strength and degrade easily as a consequence of the absence of the native-crosslinking triggered by specific enzymes present in vivo [18,23]. The weak nature of both inter- and intramolecular bonds requires exogenous crosslinking agents to obtain stable and adequate strength of the collagenous matrix, thus ensuring a proper cell adhesion and growth [24,25]. However, the most common issue related to the use of collagen crosslinking agents such as glutaraldehyde and carbodiimides concerns their limited cytocompatibility, which often induces a decrease in cell metabolic activity and adhesion [24,25,26].

In this scenario, 4-star poly(ethylene glycol) ether tetrasuccinimidyl glutarate (4-StarPEG) as a chemical crosslinking agent of collagen has been reported to increase the material stability with very low cytotoxic effects [24,26]. The study reported by Moriarty N. et al. also proved the high cytocompatibility of 4-StarPEG containing collagen hydrogels for in vivo tissue regeneration [27].

Based on one of the main objectives of the ERC BOOST project concerning the design of cell-responsive biomimetic structures for bone tissue engineering applications, in this work we developed a hybrid system combining nanostructured osteoconductive materials able to mimic the structural and compositional properties of bone and potentially stimulate cell response. In particular, the use of type I collagen and mesoporous bioactive glasses able to promote the deposition of hydroxyapatite (HA) in physiological conditions not only enables the realization of bioactive systems but also provide significant biomimetic properties with regard to the specific application. To this aim, strontium containing nano-sized MBG particles produced by a sol–gel route were incorporated in a type I collagen matrix. The peculiar sol–gel transition of the material at 37 °C was exploited to obtain a homogeneous system while the post-treatment with 4-StarPEG was explored with the aim to improve the stability of the composite without compromising its cytocompatibility. Field emission scanning electron microscope (FESEM) and energy-dispersive X-ray spectroscopy (EDS) analyses were performed to investigate the physico-chemical properties of both MBG powders and collagen hybrids and the release of strontium ions from MBG particles when included in the collagenous matrix was analyzed. The rheological characterization was conducted to determine the strength and the thermal stability of the hybrid system before and after the 4-StarPEG treatment. 

With the aim to prove the high biocompatibility of the designed formulation, MG-63 cell line adhesion to the biomaterial surface was evaluated. Immortalized cell line i.e., MG-63 is known to be a powerful tool in basic and applied biology due to its advantages in terms of availability and repeatability of results. The viability and morphology of the seeded cells were investigated at 24 and 72 h after cell seeding onto biomaterial surface by means of the Alamar Blue assay and scanning electron microscopy (SEM) respectively.

## 2. Materials and Methods 

### 2.1. Preparation of Sr Containing Sol–Gel MBG (MBG_Sr4%) 

Mesoporous bioactive glasses containing 4% molar percentage of strontium (Sr/Ca/Si = 4/11/85) were synthetized exploiting a base-catalyzed sol–gel method following the protocol previously reported by the authors [13]. Briefly, Sr-containing nanosized MBG (MBG_Sr4%) were produced dissolving 6.6 g of cetyltrimethylammonium bromide (CTAB, ≥98%, Sigma Aldrich, Milan, Italy) and 12 mL NH_4_OH (ammonium hydroxide solution, Sigma Aldrich, Milan, Italy) in 600 mL of ddH_2_O. Thirty mL of tetraethyl orthosilicate (TEOS, Tetraethyl orthosilicate, Sigma Aldrich, Milan, Italy), 4.13 g of calcium nitrate tetrahydrate (Ca(NO_3_)_2_·4H_2_O, 99%, Sigma Aldrich, Milan, Italy) and 1.68 g of strontium chloride hexahydrate (SrCl_2_·6H_2_O, for analysis EMSURE^®^ ACS) were subsequently added and the resulting suspension was kept under vigorous stirring for 3 h. After centrifugation (Hermle Labortechnik Z326, Hermle LaborTechnik GmbH, Wehingen, Germany) at 10,000 rpm for 5 min, the final precipitate was washed once with distilled water and two times with absolute ethanol, and finally dried at 70 °C for 12 h. The powders were further calcined at 600 °C in a furnace (Carbolite 1300 CWF 15/5 Carbolite Ltd., Parson Lane, Hope, Hope Valley, UK) to avoid CTAB residues.

### 2.2. Preparation of the Collagen-Based Hybrid System (Coll/MBG_Sr4%)

Type I collagen powders derived from bovine Achilles tendon (Blafar Ltd., Dublin, Ireland) were firstly dissolved in 0.5 M acetic acid stirring overnight at 4 °C. MBG_Sr4% powders were dispersed in 0.5 M acetic acid by sonicating 60 min using an ultrasonic bath (Digitec DT 103H, Bandelin, Berlin, Germany) and subsequently added to the collagen solution. The resulting suspension was stirred for 3 h to ensure optimal homogeneity. The starting amounts of collagen powders and MBG particles were calculated considering the volume percentages of the organic and inorganic phases in the natural bone tissue (53 vol. % of collagen and 47 vol. % of the inorganic phase) [28,29]. To promote the physiological reconstitution of collagen fibers, the final pH of the suspension was neutralized using 1 M NaOH reaching a final collagen concentration of 1.5 wt.%. The entire process was performed at 4 °C to avoid premature collagen crosslinking.

Exploiting the sol–gel transition of collagen at physiological pH and temperature, solid bulk samples with 10 mm diameter and 5 mm thickness were obtained by pipetting 400 μL of the suspension in a silicon mold that was subsequently incubated at 37 °C for 3 h. The samples were then collected for further processing and analyses.

### 2.3. Characterisation of MBG_Sr4% Particles 

The specific surface area (SSA) and the pore size distribution of MBG_Sr4% were investigated measuring nitrogen adsorption/desorption isotherms (ASAP2020, Micromeritics ASAP 2020 Plus Physisorption, Norcross, GA, USA) at −196 °C. In detail, SSA was measured considering the Brunauer–Emmett–Teller (BET) model while the pore size was calculated through the DFT method (density functional theory) using the NLDFT kernel of equilibrium isotherms (desorption branch). Before performing the analysis, each sample was degassed at 150 °C for 3 h. 

Morphological analyses were performed by means of field-emission scanning electron microscopy (FE-SEM) using a ZEISS MERLIN instrument (Oberkochen, Germany). The samples were prepared depositing the particles on carbon-coated copper grid (3.05 mm Diam.200 MESH, TAAB, Aldermaston, Berks, UK) and subsequently coating with 7 nm Cr layer. The same instrument was exploited to perform energy-dispersive X-ray spectroscopy (EDS) analyses in order to confirm the glass composition.

### 2.4. Characterisation of the Coll/MBG_Sr4% Hybrid System

Morphological and compositional analyses on Coll/MBG_Sr4% samples were performed to investigate the proper reconstitution of collagen fibers while observing the distribution of the glass particles embedded in the collagenous matrix. In details, Coll/MBG_Sr4% samples were frozen at −20 °C and subsequently lyophilized for 24 h using a Lyovapor L-200 freeze-dryer (Büchi, Switzerland) under vacuum (<0.1 mbar). Cross-sections of lyophilized samples were coated with a 7 nm thin chromium layer and analyzed by field-emission scanning electron microscopy (FESEM) and energy-dispersive X-ray spectroscopy (EDS). 

FTIR spectra, in the 4000–650 cm^−1^ range, were collected by using a Bruker Equinox 55 spectrometer, equipped with MCT cryodetector, at a spectral resolution of 4 cm^−1^ and accumulation of 32 scans, by using the attenuated total reflection (ATR) mode. 

### 2.5. Sr^2+^ Ion Release 

The amount of Sr^2+^ ions released from the Coll/MBG_Sr4% system was measured after soaking the samples in Tris HCl buffer (Tris(hydroxymethyl)aminomethane (Trizma, Sigma Aldrich, Milan, Italy) 0.1 M, pH 7.4) at 37 °C up to 7 days. At defined time points (10 h, 24 h, 3 days and 7 days) half of the supernatant was collected and replaced by the same volume of fresh buffer solution to keep constant the volume of the release medium. The collected supernatant was analyzed after proper dilutions by means of inductively coupled plasma atomic emission spectrometry technique (ICP-AES; ICP-MS, Thermoscientific, ICAP Q). To compare the strontium release kinetics, ICP Analyses were also performed on MBG_Sr4% powders following the procedure already described by the authors [13]. In order to calculate the final percentage of the released ions, all samples were dissolved in a mixture of nitric and hydrofluoric acids and subsequently analyzed via ICP. The analyses were conducted in triplicate and the percentages of released ions are reported as mean ± standard deviation.

### 2.6. Rheological Tests

All the rheological tests were performed using a DHR-2 controlled stress rotational rheometer (TA Instruments, Waters, Milan, Italy) equipped with parallel plate geometry with a diameter of 20 mm and a Peltier plate system to constantly control the system temperature. 

Rheological tests on 20 mm samples were conducted in order to study the strength and thermal stability of the designed material. In details, the value of storage (G’) and loss (G’’) moduli was detected by means of a dynamic amplitude sweep (0.01%–1% strain) analysis carried out at 37 °C and 1 Hz. The same samples underwent an oscillation temperature ramp (20–60 °C) under 1% strain and 1 Hz using a ramp rate of 5 °C/min in order to observe the denaturation temperature of the system associated to the sharp decrease of the visco-elastic properties. 

### 2.7. Influence of 4-StarPEG Treatment on the Coll/MBG_Sr4% Hybrid System 

#### 2.7.1. 4-StarPEG Crosslinking

With the aim to increase the material strength and stability promoting the chemical crosslinking of collagen, Coll/MBG_Sr4% samples were treated with 4-star poly (ethylene glycol) ether tetrasuccinimidyl glutarate (Abbexa Ltd., Cambridge, UK) based on procedures reported in the literature [24]. In details, bulk samples of Coll/MBG_Sr4% were immersed in 3 mL of Dulbecco phosphate buffered saline (D-PBS, Sigma Aldrich) containing 1 wt.% of 4-StarPEG at 37 °C for 24 h. After the crosslinking treatment the samples were washed two times in distilled water under mild agitation to remove any residuals of the crosslinking agent and subsequently analyzed.

#### 2.7.2. Assessment of the System Properties following 4-StarPEG Treatment 

Collagenase from *Clostridium histolyticum* (Type I, Sigma Aldrich, Milan, Italy) was used for the enzymatic degradation study before and after the 4-StarPEG crosslinking. Each sample was immersed in 1 mL of DMEM (Dulbecco’s modified eagle’s medium, Sigma Aldrich, Milan, Italy) containing 1 mg of collagenase (2.1 units) and subsequently incubated at 37 °C under static conditions. At predefined time points (12 h, 24 h and 48 h) samples were collected and abundantly washed in distilled water and then frozen. The frozen samples were then lyophilized and accurately weighted to record the weight after enzymatic digestion. After weighting lyophilized non-treated samples, weight loss was assessed using the following formula:

$$W=\left[\frac{W_{0}-W_{d}}{W_{0}}\right]\times 100$$

where $W_{0}$ represents the initial mass of the sample, $W_{d}$ represents the mass after degradation and W is the resultant loss percentage. Three samples were considered for each time point and results were reported as mean ± standard deviation.

Rheological tests were used to investigate the potential increment in material strength and thermal stability after the 4-StarPEG treatment, respectively performing amplitude sweep tests at 37 °C (0.01%–1% strain, 1 Hz) and temperature ramps (20–60 °C, 1% strain, 1 Hz) as previously reported for the non-crosslinked Coll/MBG_Sr4% system.

### 2.8. In Vitro Biological Assessment 

#### 2.8.1. MG-63 Cell Culture Expansion

MG-63 human osteosarcoma cells (ATCC) were obtained from Flow Laboratories. Cells were maintained in complete medium composed of minimum essential medium eagle—alpha modification (α-MEM; Sigma, M0644), 1% penicillin-streptomycin (Euroclone, ECB3001D; Penicillin 10.000 Units/mL, Streptomycin 10 mg/mL) and 10% fetal bovine serum—FBS (F7524Sigma, Milan, Italy) without any additional supplements in a humidified incubator at 37 °C, 95% air and 5% CO_2_. For passaging trypsin/EDTA (Trypsin 0.05%, EDTA 0.02% in PBS, Euroclone, ECB3052D) was used. The medium was refreshed every 3 days.

#### 2.8.2. MG-63 Culture on Coll/MBG_Sr4% 

At first, 200 µL of Coll/MBG_Sr4% hybrid suspension were placed into each well of a 48 well-plate in order to cover the entire bottom of the well creating a thin biomaterial layer. Once prepared, the well plates were incubated for 3 h at 37 °C and 95% humidity to enable the material gelation. Then, 1 mL of the chemical crosslinking solution consisting of 1% 4-StarPEG in PBS (phosphate buffered saline) was added to each well containing the biomaterial layer and left at 37 °C, 95% humidity for 24 h. To remove 4-StarPEG residuals, each sample was washed twice with PBS in orbital shaking for 30 min at 37 °C, 95% humidity. Before cell seeding, 2 mL of α-MEM complete culture medium were added to pre-wet each material sample for 2 h at 37 °C and 95% humidity. MG-63 cells at 80% of confluence were trypsinized and seeded at a density of 2 × 10^5^ per well (~0.95 cm^2^) on biomaterial samples. Plates were incubated at 37 °C, 95% humidity. Cell viability and adhesion were evaluated at 24 and 72 h after seeding. MG-63 cells seeded at the bottom of tissue culture polystyrene (TCPS) wells were used as controls (control).

#### 2.8.3. Cell Viability

To assess the presence of metabolically active MG-63 seeded onto biomaterial samples, the one-step Alamar Blue assay (Invitrogen, DAL1100) was performed according to the manufacturer’s instructions. Briefly, culture medium was removed and replaced with the Alamar Blue solution prepared in fresh cell culture medium (10% (v/v) Alamar Blue in culture medium) and incubated at 37 °C, 95% humidity for 3 and 5 h. Subsequently, the fluorescence of the Alamar Blue solution was quantified using a microplate reader (Infinite F200 PRO, TECAN, Mannedorf, Switzerland) at excitation and emission wavelength of 535 and 590 nm respectively. Data are expressed as a relative fluorescence unit (RFU) and reported as mean ± standard deviation of three separate experiments with quadruplicates.

#### 2.8.4. Indirect Cytotoxicity Assay using the Biomaterial-Conditioned Medium (b-CM)

To detect any potential toxic effect of the material itself when kept in complete culture medium with or without the presence of cells, an indirect test using ‘conditioned medium’ (CM) was performed. A multi-well plate containing both 4-StarPEG crosslinked and non-crosslinked biomaterial samples was prepared. A series of samples was seeded with MG-63 cells in α-MEM complete culture medium, while another series was kept with α-MEM complete culture medium only. After 24 h the medium from both series was collected to be used as biomaterial-cell conditioned medium (bc-CM) and the other as biomaterial-conditioned medium (b-CM). The two CM were applied to MG-63 cells previously seeded considering a density of 1 × 10^4^/well in a 48 well-plate. In detail, bc-CM diluted 50:50 with α-MEM, and b-CM diluted 100:0, 50:50, 25:75 and 5:95 with α-MEM were used. Viability of the cultures was assessed after an additional 24 h period.

#### 2.8.5. Cell Adhesion by Means of SEM

Samples were fixed in 2% glutaraldehyde (MERCK, 4239) in 0.1 M sodium cacodylate buffer (Sigma, C-0250) followed by washing in PBS and post-fixed in 1% osmium tetroxide (Electron Microscopy Sciences, 12310) in 0.1 M sodium cacodylate buffer. Successively, samples were dehydrated in graded alcohol series and hexamethyldisilazane and finally sputter coated with platinum (up to 7 nm thickness). Images were acquired using a Desktop SEM Phenom XL (Phenom-World B.V., The Netherlands) at an accelerating voltage of 15 kV and different magnifications.

#### 2.8.6. Statistical Analyses

Statistical analyses regarding the differences between the experimental groups and between the two time-endpoint were accomplished using the nonparametric Mann–Whitney test for unpaired data by means of the StatView 5.01 for Windows software (SAS Institute Inc., Cary, NC, USA). Results were reported as the mean ± standard deviation. Only *p* < 0.05 was considered statistically significant.

## 3. Results

### 3.1. Physico-Chemical Properties of MBG_Sr4% and Coll/MBG_Sr4%

**Figure 1:**
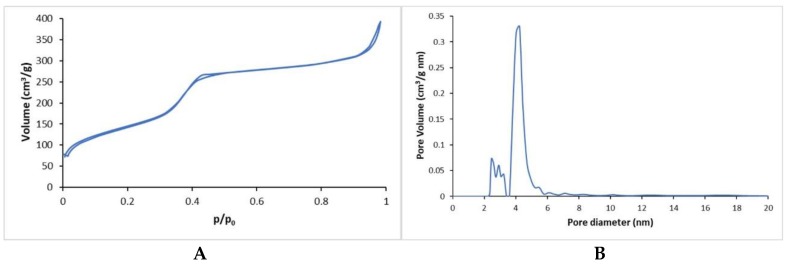
N_2_ adsorption–desorption curves of MBG_Sr4%: (**A**) isotherms and (**B**) density functional theory (DFT) pore size distributions.

In accordance with previous works reported by the authors [13,30], SiO_2_-CaO-based MBG containing strontium (4% mol.) were prepared using a base-catalyzed sol–gel route. The use of cetyltrimethylammonium bromide (CTAB) as a templating agent led to the formation of mesoporous particles as confirmed by nitrogen adsorption–desorption isotherms of MBG_Sr4% samples. The curves reported in Figure 1A were classified as type IV isotherms and were conventionally associated with mesoporous materials. As shown in Figure 1B, MBG_Sr4% particles had a uniform mesopores distribution with a mean diameter centered at about 4 nm and a small fraction of smaller pores between 2 and 4 nm. Furthermore, the analyses detected a high specific surface area (SSA) of about 528 m^2^/g and a pore volume of 0.52 cm^3^/g.

**Figure 2:**
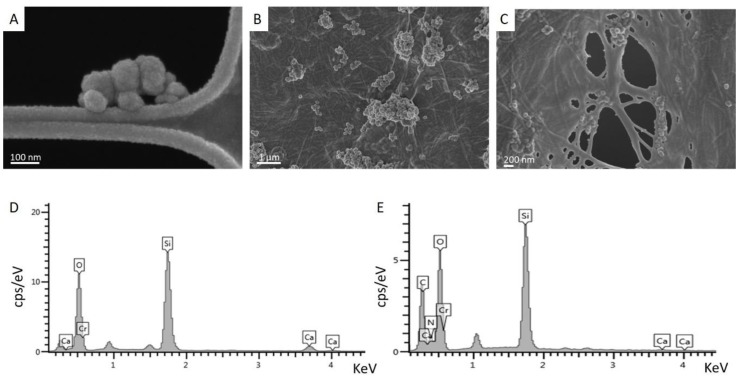
Field-emission scanning electron microscopy (FESEM) images of MBG_Sr4% particles (**A**) and Coll/MBG_Sr4% samples (**B**,**C**); energy-dispersive X-ray spectroscopy (EDS) analyses showing the composition of MBG_Sr4% (**D**) and Coll/MBG_Sr4% system (**E**).

FESEM images of MBG_Sr4% samples (Figure 2A) proved the formation of spherical particles with a nano-size ranging from 50 nm up to 200 nm. 

The obtained nanoparticles were subsequently incorporated in a type I collagen matrix resulting in a homogeneous hybrid system. The physical crosslinking of collagen triggered by the simil-physiological condition of pH and temperature (7.4; 37 °C) led to the reconstitution of fibrous wall and meshes homogeneously embedding the inorganic phase. Despite the typical tendency of nano-sized particles to agglomerate, MBG_Sr4% appeared rather well distributed and formed clusters less than few microns in size, as shown in Figure 2B,C. 

Energy-dispersive X-ray spectroscopy (EDS) analyses confirmed the composition of the glass particles based on silica (Si, O) and calcium oxide (Ca, O), as shown in Figure 2D. Analyses performed on Coll/MBG_Sr4% samples proved the large presence of glass particles in the system while showing the appearance of C and N peaks related to the chemical composition of collagen.

The reported results were supported and confirmed also by means of ATR-FTIR spectroscopy (Figure S1) where the characteristic peaks related to both type I collagen and MBG_Sr4% were detected and mostly evident in the 1800–800 cm^−1^ range.

### 3.2. Sr^2+^ Ion Release 

**Figure 3:**
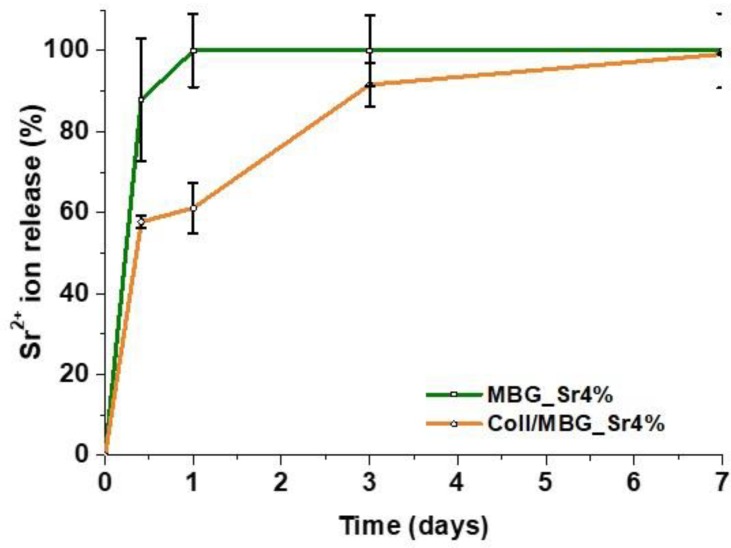
Sr^2+^ ion release from MBG_Sr4% particles and the Coll/MBG_Sr4% system.

The released concentrations of Sr^2+^ ions in Tris-HCl buffer from both MBG_Sr4% and Coll/MBG_Sr4% were determined by means of ICP analyses up to 7 days and compared. The percentage of ion released was calculated considering the amount of strontium initially incorporated in MBG particles (as measured by ICP on acid-digested samples) and the content of inorganic phase incorporated into the hybrid system. As shown in Figure 3, the released percentage was lower when MBG particles were incorporated into the collagenous matrix: about 87% and 57% of ion release after 10 h from MBG_Sr4% and Coll/MBG_Sr4%, respectively. From particles alone, the entire amount of strontium content was delivered within 24 h, on the contrary, the hybrid system showed more sustained release kinetics, reaching the 100% after 7 days of incubation in Tris-HCl buffer.

### 3.3. Coll/MBG_Sr4% System Strength and Stability

**Figure 4:**
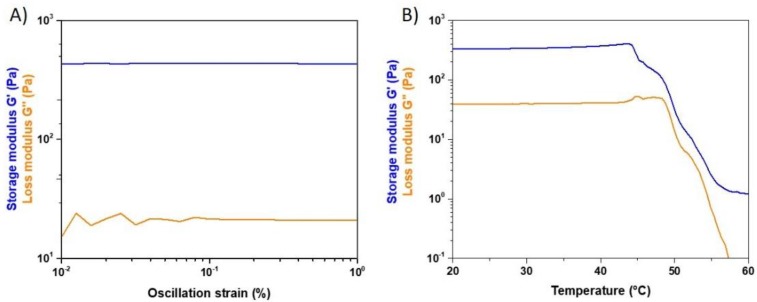
Amplitude sweep test (**A**) and temperature ramp (**B**) performed on Coll/MBG_Sr4% samples.

The mechanical and thermal stability of the hybrid system was investigated by means of rheological tests. In particular, amplitude sweep tests performed at 37 °C identified the linear viscoelastic region of the material between 0.01% and 1% strain at a constant frequency of 1 Hz. As shown in Figure 4A, the average values of storage modulus (G’) and loss modulus (G’’) in the selected range of strains varied between 430 and 480 Pa and 46 and 65 Pa respectively. The relevant gap between G’ and G’’ proved the solid-like nature of the samples obtained after the sol–gel transition of the material at 37 °C.

Coll/MBG_Sr4% samples subjected to a temperature ramp performed between 20 and 60 °C (Figure 4B) reported a sharp decrease of both storage and loss modulus at about 44.7 °C, mainly related to the denaturation of collagen and the subsequent loss of the structural and mechanical stability of the material.

### 3.4. Effects of 4-StarPEG on Coll/MBG_Sr4% System

Coll/MBG_Sr4% system was treated with 4-StarPEG chemical crosslinker with the aim to improve the mechanical and thermal stability of the material while maintaining high biocompatibility.

To this aim, Coll/MBG_Sr4% samples were incubated in 4-StarPEG solution at 37 °C for 24 h in order to allow the crosslinker diffusion and the further reaction with collagen functional groups.

After the crosslinking treatment, samples were analyzed by means of rheological tests to investigate the resulting mechanical and thermal strength. The biomaterial was subjected to amplitude sweep tests and temperature ramps as previously reported for non-treated system. Results (reported in Figure S2) showed an increase in the storage and loss modulus with values in the range of 617–741 Pa and 50–65 Pa respectively. On the contrary, no differences were detected in the denaturation temperature of the material, with a resulting drop of G’ and G’’ at 44.7 °C.

**Figure 5:**
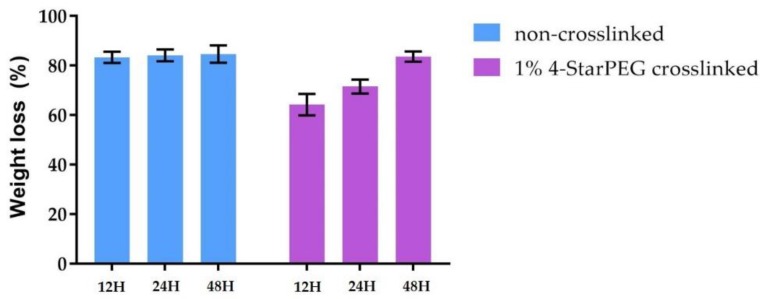
Weight loss of 4-StarPEG crosslinked and non-crosslinked Coll/MBG_Sr4% samples following incubation in collagenase up to 48 h.

To further investigate the material stability, the degradation of 4-StarPEG crosslinked and non-crosslinked samples in a concentrated collagenase solution was compared. As represented in Figure 5, the investigated samples lost most of their initial weight after 48 h when subjected to collagenase digestion. Even though 4-StarPEG crosslinked Coll/MBG_Sr4% samples retained about 60% of the initial weight at 12 h of incubation, the detected degradation rate proved a poor effect of the chemical crosslinker on the material stability.

### 3.5. Cell Viability

**Figure 6:**
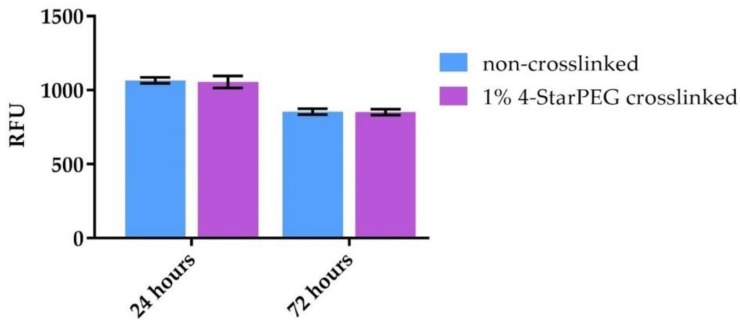
MG-63 cell viability on 4-StarPEG crosslinked and non-crosslinked samples at 24 and 72 h using the Alamar Blue assay.

Figure 6 shows the results of cell viability using the Alamar Blue assay. 4-StarPEG crosslinked Coll/MBG_Sr4% samples were compared to the non-crosslinked system in order to discriminate the biocompatibility of the designed system and the crosslinking treatment. The background value of biomaterial without cells seeded, recorded during the reading of the assay, was extremely low (69–94 RFU) and was subtracted from the data. Control of MG-63 was performed and analyzed at 24 h and the value was 694 RFU. No relevant differences in terms of viability were observed between chemically crosslinked and non-crosslinked samples. Even though no proliferation was observed, cell viability was maintained after 72 h of culture. Statistical analyses revealed that no significant statistical differences in cell viability were observed between non-treated and treated biomaterial samples. 

**Figure 7:**
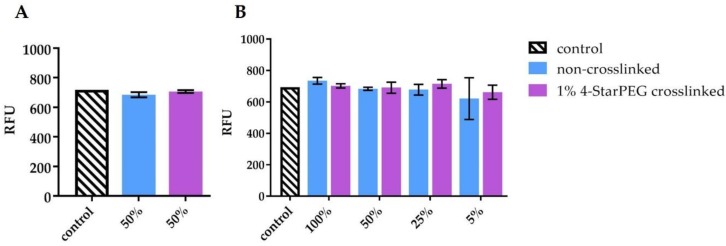
MG-63 cell viability indirect assay, using the conditioned medium (CM) diluted 1:1 (**A**) and 100:0, 50:50, 25:75 and 5:95 with fresh culture medium (**B**).

Figure 7 shows the MG-63 cell viability at 24 h using the conditioned medium (CM) from 4-StarPEG crosslinked and non-crosslinked samples, cultured for 24 h with (bc-CM, Figure 7A) or without (b-CM, Figure 7B) MG-63 cells. In particular, bc-CM was diluted 50:50 with culture medium (Figure 7A) while b-CM was diluted 100:0, 50:50, 25:75 and 5:95 with culture medium (Figure 7B). Viability of MG-63 cells challenged with the two types of CM was unaffected, confirming the absence of any toxic substance released from the hybrid systems. 

### 3.6. Cell Adhesion by Means of Scanning Electron Microscopy (SEM) Analyses

**Figure 8:**
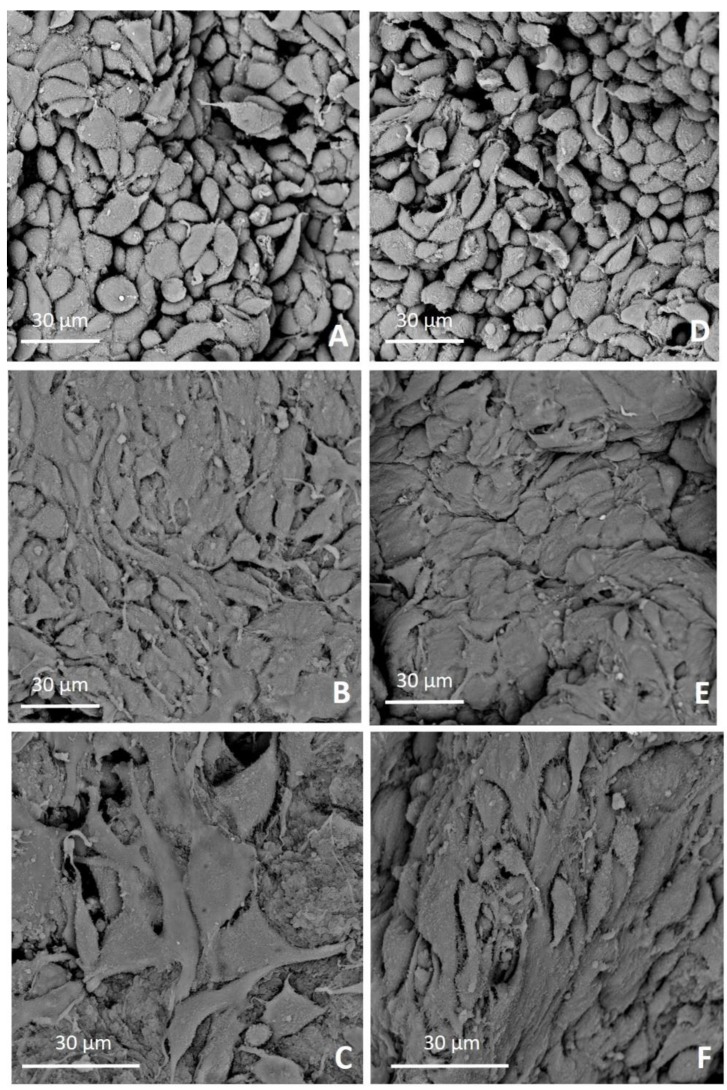
SEM microphotographs of MG-63 adhesion on 4-StarPEG crosslinked Coll/MBG_Sr4% at 24 h (**A**) and 72 h (**B**,**C**), and non-crosslinked Coll/MBG_Sr4% after 24 h (**D**) and 72 h (**E**,**F**).

SEM analyses were performed to evaluate the adhesion and morphology of MG-63 on both 4-StarPEG crosslinked and non-crosslinked Coll/MBG_Sr4% hybrid system at 24- and 72-h after seeding. SEM microphotographs showed a good adhesion of MG-63 on the biomaterial surfaces at both time endpoints. At 24 h a significant number of cells adhered to the biomaterial surface exhibiting a cuboidal shape (Figure 8A,D), while at 72 h the cells strongly adhered and easily spread showing a flat and elongated shape (Figure 8B,C,E,F).

Prominent filopodia and extracellular protrusions were observed to connect the cells each other and between cells and biomaterial surfaces at 72 h, especially on 4-StarPEG crosslinked Coll/MBG_Sr4% (Figure 8C). Cells on the 4-StarPEG crosslinked samples appeared spread and more stretched (Figure 8C) than the ones on the non-crosslinked biomaterial (Figure 8F).

Cell morphology observed on biomaterial samples were compared to the l MG-63 control. In detail, MG-63 only were seeded onto standard TCPS 24-well plate at two different densities: 1 × 10^4^ cells/well (Figure S3), and 2 × 10^5^ cells/well (Figure S4) and observed under scanning electron microscope (SEM) at 24 h from seeding.

## 4. Discussion

Numerous studies have reported the great potential of mesoporous bioactive glasses in bone tissue engineering applications strongly related to their ability to promote hydroxyapatite deposition and the further formation of high-strength interface between natural tissue and biomaterial [11]. Among the large variety of bioactive glasses in terms of morphology, size and composition, in the present work, Sr-containing (4% mol) nano-sized MBG particles were selected as promising candidates due to the significant bioactive and pro-osteogenic properties recently reported by the authors [13]. The inclusion of bioactive glasses in polymeric matrices leads to the creation of hybrid systems that widen their field of application, enabling the design of biomimetic scaffolds while improving the interface between cells and material [11,31,32].

In this frame, the selection of type I collagen as polymeric phase represents a key factor in view of designing biomimetic systems for bone regeneration treatments. Representing the main organic phase of bone, type I collagen is able to reconstitute a complex fibrous architecture in physiological conditions while providing specific cell binding sites, overall improving the interaction with cells [21,33].

The successful inclusion of MBG_Sr4% in type I collagen was proved by means of FESEM and EDS analyses, while nitrogen adsorption–desorption isotherms showed the significant specific surface area and the mesoporous nature of glass particles, responsible for the well-known high bioactive properties [13].

FESEM images clearly reported the homogenous distribution of the spherical nano-sized MBG particles into the collagen matrix following the sol–gel transition of the material at 37 °C. The morphological evaluation thus proved that the self-assembly of collagen fibers was not hampered by the presence of MBG particles, leading to final well-packed nanofibril networks in the Coll/MBG_Sr4% hybrid system. 

The composition of the bioactive glasses was confirmed by EDS spectra, whereas the presence of strontium was not detectable due to the overlapping with the predominant silicon peaks. However, the strontium content was detected by ICP analyses after sample acidic digestion. In addition, the ability of the designed system to deliver pro-osteogenic Sr^2+^ ions was evidenced by ICP analyses on both MBG particles and Coll/MBG_Sr4% samples. The presence of the collagenous matrix enabled a more sustained release of the ions, dampening the burst release observed for MBG_Sr4% alone. A more sustained release of functional ions is highly desirable in order to extend the pro-osteogenic effect of the biomaterial. In a recent work, Naruphontjirakul P. and coworkers proved that Sr-containing bioactive glass nanoparticles were able not only to influence osteoblastic cell activity but also to guide the osteogenic differentiation of bone marrow derived human stem cells. Furthermore, the 4% mol. concentration has been selected considering that higher concentrations did not lead to additional benefit as previously reported [17].

Besides the structural and physico-chemical characterization of the formulated hybrid system, rheological tests were performed to investigate its mechanical and thermal stability. The strength of the material was assessed observing the values of the elastic and storage modulus at 37 °C while the effect of temperature increase was observed up to 60 °C. 

The viscoelastic properties of the system were clearly represented by the resulting pattern of G’ and G’’, where the greater contribution of the elastic modulus defined the solid-like nature of the material, despite the significant amount of water. Coll/MBG_Sr4% samples exposed to increasing temperatures showed stable values of G’ and G’’ up to about 45 °C, proving their stability at body temperature. The following drop in the material mechanical properties was related to the denaturation of collagen, where the high temperature promoted the break of the weak inter- and intra-molecular bonds [34]. 

Despite the proved solid nature of the material, the relatively low values of complex modulus measured and the weak physical bonds between collagen molecules suggested the need of an additional chemical treatment to create intermolecular covalent bonds able to improve the final strength and stability of the biomaterial. Furthermore, as cells recognize the elasticity of the surrounding substrate and differentiate accordingly, hybrid systems with higher stiffness are considered to provide better 3D conditions for osteogenic differentiation [24,35,36].

The main limitation in the use of chemical crosslinkers is represented by their often-reported cytotoxic effect, which leads to the overall decrease of the system biocompatibility, restricting the use of the final material [24,37]. For this reason, 4-star poly (ethylene glycol) ether tetrasuccinimidyl glutarate (4-StarPEG) was selected as potential collagen chemical crosslinker due to the high cytocompatibility previously proved considering both in vitro and in vivo tests [26,27]. Accordingly, in the study conducted by Moriarty N. et al., 4-StarPEG was successfully incorporated in the formulation of hydrogel spheres for cell encapsulation without hindering cell metabolic activity and vitality [27]. In detail, the crosslinking process involves the reaction between the succinimidyl groups of 4-StarPEG with the amine groups present on the collagen molecules and the further formation of intermolecular strong covalent bonds [24].

Rheological and collagenase degradation tests were exploited to compare the properties of the hybrid system before and after the 4-StarPEG treatment to detect any potential increase in its mechanical and thermal stability. However, the differences observed comparing the two systems are not so significant. In details, G’ increased of more than 50% whereas G’’ was almost stable after the crosslinking treatment; the denaturation temperature of the material was measured at about 45 °C, as previously obtained for the non-treated Coll/MBG_Sr4% samples. Similarly, the collagenase digestion led to almost the complete degradation of the material in 48 h of incubation even after the crosslinking process, although the degradation kinetics shown by crosslinked Coll/MBG_Sr4% were slower. The reported results thus suggested an overall low efficiency of the selected crosslinking method, probably linked to low diffusion kinetics in the water-containing polymeric matrix, whereas higher crosslinking efficiency was obtained when using 4-StarPEG on lyophilized matrices. In such cases, the authors reported high resistance to enzymatic degradation in addition to a significant reduction of free amino content after 4-starPEG crosslinking, proving the effective enhancement of the material stability [24,26]. However, the mechanical strength of 4-StarPEG crosslinked collagen resulted to be weaker when compared to matrices crosslinked with alternative methods such as genipin and glutaraldehyde [24,38].

Despite the low increase in material stability, we proved the high cytocompatibility of the designed hybrid system before and after the treatment with 4-StarPEG. The biological assessment was performed in presence of MG-63 cell line, observing both viability and adhesion of the cells cultured onto the biomaterial.

The use of immortalized cell lines is more frequent than primary bone cells in the field of applied biology and bone tissue engineering research due to the availability of unlimited amount of cells and more reliable reproducibility by replicate experiments [39,40]. MG-63 cell line has been defined both as the immature osteoblast phenotype model [41] and pre-osteoblastic model [42] and for this reason different authors observed that MG-63 cell type displays a heterogeneous behavior [43]. This cell model is one of the most used for cytocompatibility studies and it is extremely useful in the first step (pre-screening) of biomaterial evaluation [44]. In order to perform the biological assessment of the designed hybrid system we focused on the two main features for initial assay of biomaterials, represented by cell adhesion and cell viability. The Alamar Blue assay is widely used to evaluate cell viability and cytotoxicity of agents [45]. To assess the performance of MG-63 seeded onto biomaterial surface, two endpoints were selected: cell adhesion was checked at 24 h, while cell viability and the potential cell proliferation at 72 h. In both systems, i.e., non-crosslinked and 4-StarPEG crosslinked Coll/MBG_Sr4% group, cell viability was maintained over time, confirming that the hybrid suspension was biocompatible. Moreover, our data indicated that the chemical crosslinking reaction by means of 4-StarPEG was not toxic for cells and the crosslinked samples hosted viable cells.

Since the absence of cell proliferation at 72 h was observed in both non-crosslinked and crosslinked system, we could conclude that it was not related to the crosslinking procedure or potential residuals.

In order to provide further insights about the potential cytotoxic effect of released or degradation sub-products from the hybrid system, an indirect cytotoxicity assay was performed with two different variants of conditioned medium (CM). In one case CM derived from the contact with the biomaterial only (b-CM) and in the other case, the CM derived from the contact with the biomaterial previously seeded with cells (bc-CM). The two conditioned media were thus separately tested on MG-63 layers for 24 h. The reported method allowed to better understand if the biomaterial released any toxic substances after simple storage in the culture medium or if the presence of cells onto the biomaterial could influence or accelerate such a release in the medium. From collected data, it is possible to state that the biomaterial did not release toxic substances since the cell viability with b-CM was maintained over time for any tested dilution, from the most concentrated one (100% CM) to the most diluted one (5% CM, 5:95 with fresh culture medium). Moreover, the presence of cells seeded onto the biomaterial (bc-CM) did not induce the formation and/or the release of toxic components/substances, since MG-63 viability was unaltered after 24 h. 

We further investigated the interaction between both 4-StarPEG crosslinked and non-crosslinked system and cells observing the morphology of adherent cells by means of SEM microscopy. In detail, MG-63 cells firmly attached to the material surfaces and kept this behavior over time for both 4-StarPEG crosslinked and non-crosslinked samples. Furthermore, cells seeded on 4-StarPEG crosslinked samples seemed to be more flattened and stretched, even if the stiffness and mechanical strength of the biomaterial was only slightly higher than the non-crosslinked one. SEM analyses suggested that the hybrid system had a positive interaction with MG-63 cells promoting a consistent cell adhesion while enabling the maintenance of cell viability over time. 

All the presented data highlighted not only the positive effects on cell response but clearly showed the higher potential of the hybrid system compared to the use of bioactive glass particles alone in the bone tissue engineering field. The area of application can be further extended considering the peculiar sol–gel transition of the material. In details, the hybrid suspension can be printed exploiting the freeform reversible embedding of suspended hydrogels as reported by Hinton et al. [46], with the aim to create more complex bone-like structures able to release therapeutic ions.

## 5. Conclusions

Pro-osteogenic mesoporous bioactive glass particles were successfully and homogeneously incorporated in type I collagen fibrous matrix, forming a biomimetic hybrid system in physiological conditions. The designed formulation was able to release Sr^2+^ strontium ions up to 7 days with a more sustained kinetics compared to the MBG particles alone. The system proved mechanical stability at body temperature, whereas denaturation processes were detected for temperatures higher than 44 °C. Despite the promising results reported in the literature, no substantial improvement in the mechanical and thermal stability of the material was detected upon treatment with 4-StarPEG as cross-linking agent, suggesting the need for the selection of alternative approaches in future works. Nevertheless, both 4-StarPEG crosslinked and non-crosslinked samples showed high biocompatibility promoting good MG-63 cell viability and adhesion. Due to the reported promising properties of the developed system and the peculiar sol–gel transition at 37 °C, future studies may consider it for 3D extrusion printing applications to design biomimetic scaffolds for bone tissue regeneration. 

## Supplementary Materials

The following are available online at https://www.mdpi.com/1996-1944/12/22/3719/s1, Figure S1: ATR-FTIR of Coll/MBG_Sr4% hybrid system, Figure S2: Amplitude sweep test (A) and temperature ramp (B) performed on 4-StarPEG crosslinked Coll/MBG_Sr4% samples, Figure S3: Morphology of control MG-63 seeded at 1 × 10^4^ onto standard TCPS 24-well plate and observed by SEM at 24 h. Scale bars are shown, Figure S4: Morphology of control MG-63 seeded at 2 × 10^5^ onto standard TCPS 24-well plate and observed by SEM at 24 h. Scale bars are shown.

## References

[1] Miri A.K., Muja N., Kamranpour N.O., Lepry W.C., Boccaccini A.R., Clarke S.A., Nazhat S.N. (2016). Biomaterials Ectopic bone formation in rapidly fabricated acellular injectable dense collagen-Bioglass hybrid scaffolds via gel aspiration-ejection. Biomaterials.

[2] Sterling J.A., Guelcher S.A. (2014). Biomaterial scaffolds for treating osteoporotic bone. Curr. Osteoporos. Rep..

[3] Arcos D., Boccaccini A.R., Bohner M., Díez-pérez A., Epple M., Gómez-barrena E. (2014). The relevance of biomaterials to the prevention and treatment of osteoporosis. Acta Biomater..

[4] Roseti L., Parisi V., Petretta M., Cavallo C., Desando G., Bartolotti I., Grigolo B. (2017). Scaffolds for Bone Tissue Engineering: State of the art and new perspectives. Mater. Sci. Eng. C.

[5] Tang D., Tare R.S., Yang L.Y., Williams D.F., Ou K.L., Oreffo R.O.C. (2016). Biofabrication of bone tissue: Approaches, challenges and translation for bone regeneration. Biomaterials.

[6] Campana V., Milano G., Pagano E., Barba M., Cicione C., Salonna G., Lattanzi W., Logroscino G. (2014). Bone substitutes in orthopaedic surgery: From basic science to clinical practice. J. Mater. Sci. Mater. Med..

[7] Jakob F., Ebert R., Ignatius A., Matsushita T., Watanabe Y., Groll J., Walles H. (2013). Bone tissue engineering in osteoporosis. Maturitas.

[8] Glowacki J., Mizuno S. (2007). Collagen scaffolds for tissue engineering. Biopolymers.

[9] Patel A., Mequanint K. (2007). Hydrogel biomaterials. Biomaterials.

[10] Basha R.Y., Doble M. (2015). Design of biocomposite materials for bone tissue regeneration. Mater. Sci. Eng. C.

[11] Sarker B., Hum J., Nazhat S.N., Boccaccini A.R. (2015). Combining collagen and bioactive glasses for bone tissue engineering: A review. Adv. Healthcare Mater..

[12] Rezwan K., Chen Q.Z., Blaker J.J., Roberto A. (2006). Biodegradable and bioactive porous polymer/inorganic composite scaffolds for bone tissue engineering. Biomaterials.

[13] Fiorilli S., Molino G., Pontremoli C., Iviglia G., Torre E., Cassinelli C., Morra M., Vitale-Brovarone C. (2018). The incorporation of strontium to improve bone-regeneration ability of mesoporous bioactive glasses. Materials.

[14] Naruphontjirakul P., Porter A.E., Jones J.R. (2018). In vitro osteogenesis by intracellular uptake of strontium containing bioactive glass nanoparticles. Acta Biomater..

[15] Zhang Y., Cui X., Zhao S., Wang H., Rahaman M.N., Liu Z., Huang W., Zhang C. (2015). Evaluation of Injectable Strontium-Containing Borate Bioactive Glass Cement with Enhanced Osteogenic Capacity in a Critical-Sized Rabbit Femoral Condyle Defect Model. Mater. Interfaces.

[16] Taherkhani S., Moztarzadeh F. (2016). Influence of strontium on the structure and biological properties of sol–gel-derived mesoporous bioactive glass (MBG) powder. J. Sol-Gel Sci. Technol..

[17] Naruphontjirakul P., Tsigkou O., Li S., Porter A.E., Jones J.R. (2019). Human mesenchymal stem cells differentiate into an osteogenic lineage in presence of strontium containing bioactive glass nanoparticles. Acta Biomater..

[18] Turnbull G., Clarke J., Picard F., Riches P., Jia L., Han F., Li B., Shu W. (2018). 3D bioactive composite scaffolds for bone tissue engineering. Bioact. Mater..

[19] Khatami N. (2019). Collagen—Alginate—Nano—Silica microspheres improved the osteogenic potential of human osteoblast—Like MG—63 cells. J. Cell. Biochem..

[20] Kumar A., Rao K.M., Han S.S. (2017). Synthesis of mechanically stiff and bioactive hybrid hydrogels for bone tissue engineering applications. Chem. Eng. J..

[21] Ferreira A.M., Gentile P., Chiono V., Ciardelli G. (2012). Collagen for bone tissue regeneration. Acta Biomater..

[22] Angele P., Abke J., Kujat R., Faltermeier H., Schumann D., Nerlich M., Kinner B., Englert C., Ruszczak Z., Mehrl R. (2004). Influence of different collagen species on physico-chemical properties of crosslinked collagen matrices. Biomaterials.

[23] Ward J., Kelly J., Wang W., Zeugolis D.I., Pandit A. (2010). Amine Functionalization of Collagen Matrices with Multifunctional Polyethylene Glycol Systems. Biomacromolecules.

[24] Delgado L.M., Fuller K., Zeugolis D.I. (2017). Collagen Cross-Linking: Biophysical, Biochemical, and Biological Response Analysis. Tissue Eng. Part A.

[25] Madhavan K., Belchenko D., Motta A., Tan W. (2010). Evaluation of composition and crosslinking effects on collagen-based composite constructs. Acta Biomater..

[26] Sanami M., Shtein Z., Meirovich S., Sorushanova A., Mullen A.M., Miraftab M., Shoseyov O., Dowd C.O., Pandit A., Zeugolis D.I. (2015). The influence of poly (ethylene glycol) ether tetrasuccinimidyl glutarate on the structural, physical, and biological properties of collagen fibers. J. Biomed. Mater. Res. B Appl. Biomater..

[27] Moriarty N., Pandit A., Dowd E. (2017). Encapsulation of primary dopaminergic neurons in a GDNF—Loaded collagen hydrogel increases their survival, re-innervation and function after intra-striatal transplantation. Nat. Sci. Rep..

[28] Montalbano G., Fiorilli S., Caneschi A., Vitale-brovarone C. (2018). Type I Collagen and Strontium-Containing Mesoporous Glass Particles as Hybrid Material for 3D Printing of Bone-Like Materials. Materials.

[29] Schwarcz H.P., Abueidda D., Jasiuk I. (2017). The Ultrastructure of Bone and Its Relevance to Mechanical Properties. Front. Phys..

[30] Pontiroli L., Dadkhah M., Novajra G., Tcacencu I., Fiorilli S., Vitale-brovarone C. (2017). An aerosol-spray-assisted approach to produce mesoporous bioactive glass microspheres under mild acidic aqueous conditions. Mater. Lett..

[31] Sharifi E., Azami M., Kajbafzadeh A., Moztarzadeh F., Faridi-majidi R., Shamousi A., Karimi R., Ai J. (2016). Preparation of a biomimetic composite scaffold from gelatin/collagen and bioactive glass fi bers for bone tissue engineering. Mater. Sci. Eng. C.

[32] Eglin D., Maalheem S., Livage J., Coradin T. (2006). In vitro apatite forming ability of type I collagen hydrogels containing bioactive glass and silica sol-gel particles. J. Mater. Sci. Mater. Med..

[33] Sherman V.R., Yang W., Meyers M.A. (2015). The materials science of collagen. J. Mech. Behav. Biomed. Mater..

[34] Walters B.D., Stegemann J.P. (2014). Strategies for directing the structure and function of three-dimensional collagen biomaterials across length scales. Acta Biomater..

[35] El-fiqi A., Ho J., Lee E., Kim H. (2013). Collagen hydrogels incorporated with surface-aminated mesoporous nanobioactive glass: Improvement of physicochemical stability and mechanical properties is effective for hard tissue engineering. Acta Biomater..

[36] Marelli B., Ghezzi C.E., Barralet J.E., Boccaccini A.R., Nazhat S.N. (2010). Three-Dimensional Mineralization of Dense Nanofibrillar Collagen—Bioglass Hybrid Scaffolds. Biomacromolecules.

[37] Davidenko N., Schuster C.F., Bax D.V., Raynal N., Farndale R.W., Best S.M., Cameron R.E. (2015). Control of crosslinking for tailoring collagen-based scaffolds stability and mechanics. Acta Biomater..

[38] Zhang X., Chen X., Yang T., Zhang N., Dong L., Ma S., Liu X., Zhou M., Li B. (2014). The effects of different crossing-linking conditions of genipin on type I collagen scaffolds: An in vitro evaluation. Cell. Tissue Bank..

[39] Czekanska E.M., Stoddart M.J., Ralphs J.R., Richards R.G., Hayes J.S. (2014). A phenotypic comparison of osteoblast cell lines versus human primary osteoblasts for biomaterials testing. J. Biomed. Mater. Res. Part A.

[40] Kartsogiannis V., Ng K.W. (2004). Cell lines and primary cell cultures in the study of bone cell biology. Mol. Cell. Endocrinol..

[41] Czekanska E., Stoddart M., Richards R., Hayes J. (2012). In search of an osteoblast cell model for in vitro research. Eur. Cells Mater..

[42] Saldaña L., Bensiamar F., Boré A., Vilaboa N. (2011). In search of representative models of human bone-forming cells for cytocompatibility studies. Acta Biomater..

[43] Pautke C., Schieker M., Tischer T., Kolk A., Neth P., Mutschler W., Milz S. (2004). Characterization of osteosarcoma cell lines MG-63, Saos-2 and U-2 OS in comparison to human osteoblasts. Anticancer Res..

[44] Burova I., Peticone C., Thompson D.D.S., Knowles J.C., Wall I., Shipley R.J. (2019). A parameterised mathematical model to elucidate osteoblast cell growth in a phosphate-glass microcarrier culture. J. Tissue Eng..

[45] Bonnier F., Keating M.E., Wróbel T.P., Majzner K., Baranska M., Garcia-Munoz A., Blanco A., Byrne H.J. (2015). Cell viability assessment using the Alamar blue assay: A comparison of 2D and 3D cell culture models. Toxicol. Vitr..

[46] Hinton T.J., Jallerat Q., Palchesko R.N., Park H.J., Grodzicki M.S., Shue H.-J., Ramadan H.M., Hudson A.R., Feinberg A.W. (2015). Three-dimensional printing of complex biological structures by freeform reversible embedding of suspended hydrogels. Sci. Adv..

