# Supplementary material for: Development and Biocompatibility of Collagen-Based Composites Enriched with Nanoparticles of Strontium Containing Mesoporous Glass
Source: Materials (Basel). 2019 Nov 11;12(22):3719. doi: 10.3390/ma12223719 (PMC6888293; doi:10.3390/ma12223719)
Supplement: Supplementary file 1 [file materials-12-03719-s001.pdf]

materials

Supporting Information

Development and Biocompatibility of Collagen-based Composites enriched with Nanoparticles of Strontium containing Mesoporous Glass

Giorgia Montalbano [1], Giorgia Borciani [1,2], Carlotta Pontremoli [1], Gabriela Ciapetti [2], Monica Mattioli-Belmonte [3], Sonia Fiorilli [1] and Chiara Vitale-Brovarone [1,*]

[1] Applied Science and Technology Department, Politecnico di Torino, Corso Duca degli Abruzzi 24, 10129, Torino, Italy; giorgia.montalbano@polito.it (G.M.); giorgia.borciani@polito.it (G.B.); carlotta.pontremoli@polito.it (C.P.); sonia.fiorilli@polito.it (S.F.)

[2] Laboratorio di Fisiopatologia Ortopedica e Medicina Rigenerativa, IRCCS Istituto Ortopedico Rizzoli, 40136, Bologna, Italy; gabriela.ciapetti@ior.it

[3] Dipartimento di Scienze Cliniche e Molecolari, DISCLIMO, Università Politecnica delle Marche, 60100, Ancona, Italy; m.mattioli@univpm.it

* Correspondence: chiara.vitale@polito.it; Tel.: +39-0110-904-716

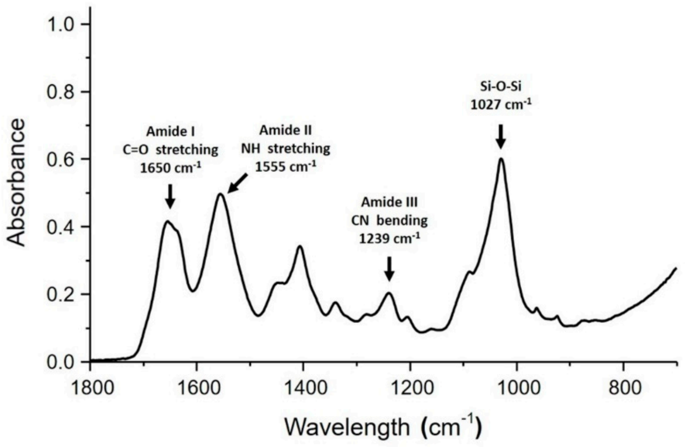

Figure S1. ATR-FTIR of Coll/MBG_Sr4% hybrid system.

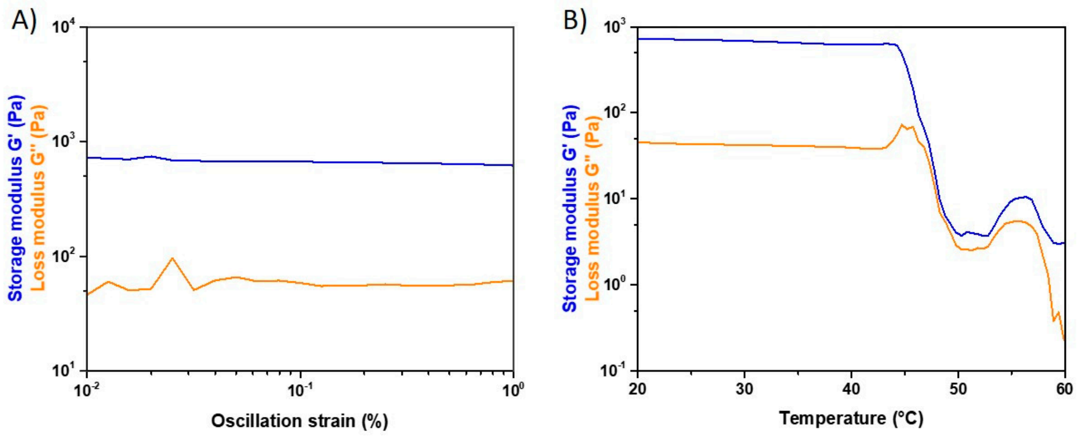

Figure S2. Amplitude sweep test (**A**) and temperature ramp (**B**) performed on 4-StarPEG crosslinked Coll/MBG_Sr4% samples.

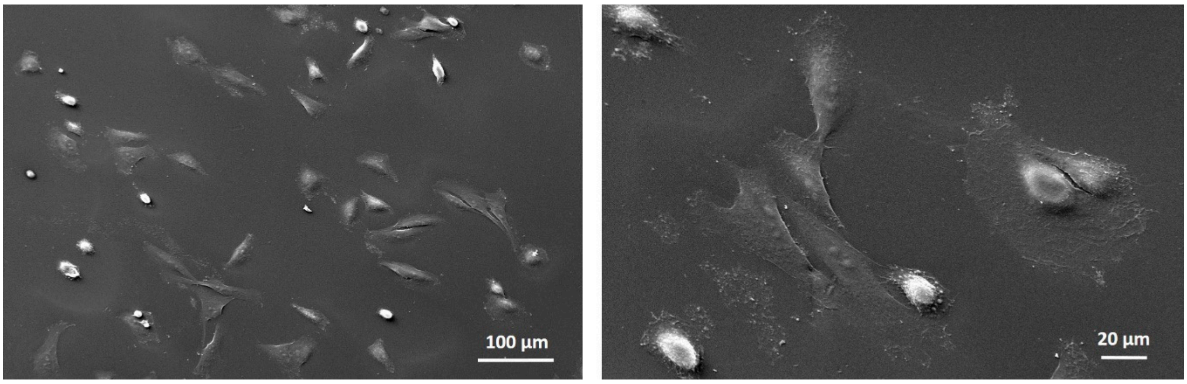

Figure S3. Morphology of control MG-63 seeded at 1 × 10^4 onto standard TCPS 24-well plate and observed by SEM at 24 h. Scale bars are shown.

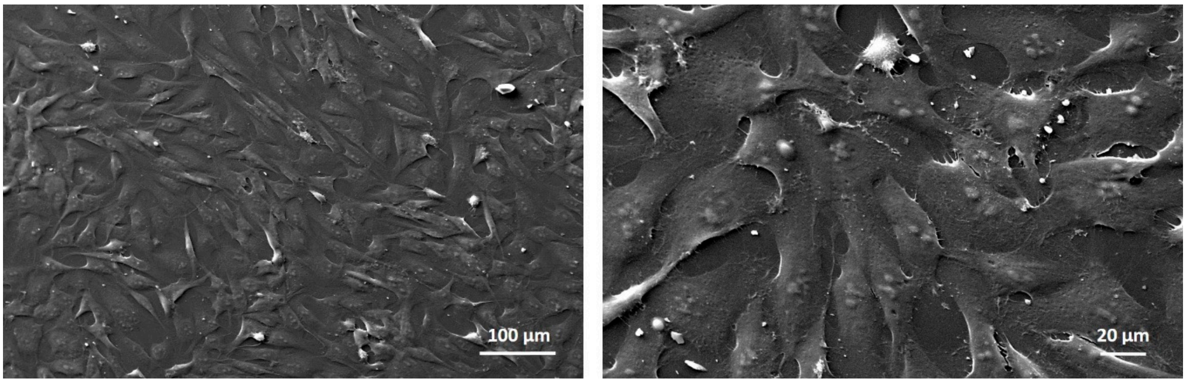

Figure S4. Morphology of control MG-63 seeded at 2 × 10^5 onto standard TCPS 24-well plate and observed by SEM at 24 h. Scale bars are shown.
